# Supplementary material for: Lockdown, relaxation, and acme period in COVID-19: A study of disease dynamics in Hermosillo, Sonora, Mexico
Source: PLoS One. 2020 Dec 3;15(12):e0242957. doi: 10.1371/journal.pone.0242957 (PMC7714188; doi:10.1371/journal.pone.0242957)
Supplement: S1 File — (PDF) [file pone.0242957.s001.pdf]

Supplementary Material
Lockdown, relaxation, and acme period in COVID-19: A study of disease dynamics in Hermosillo, Sonora, Mexico

Mayra R. Tocto-Erazo[1], Jorge A. Espíndola-Zepeda[1], José A. Montoya-Laos[1], Manuel A. Acuña-Zegarra[1], Daniel Olmos-Liceaga[1], Pablo A. Reyes-Castro[2], Gudelia Figueroa-Preciado[1*]

1 Departamento de Matemáticas, Universidad de Sonora, Blvd. Luis Encinas y Rosales S/N, Hermosillo, Sonora, C.P. 83000, México.
2 Centro de Estudios en Salud y Sociedad, El Colegio de Sonora, Av. Obregón 54, Hermosillo, Sonora, C.P. 83000, México.

S1 About the acme occurring time

In the main document, for each one of the scenarios, we showed the behavior of daily new reported cases. Here, S1 Fig illustrates the behavior of daily new hospitalizations and daily new deaths. Similar to Fig 3, included in the main document, we can observe that each scenario shows a good fit to the data, although it is noticeable a lot of variability in their respective acme levels, reinforcing the identifiability parameter problem mentioned in the main document.

S2 Implications of Lockdown occurrence time

To complement the information presented in Fig 6 included in the main document, here S2 Fig shows, for Scenarios 1 and 3, the possible consequences of having a same period lockdown, but with one or two weeks delay. Observe that in accordance with Scenario 2, there is a considerable increase in the prevalence of hospitalized and cumulative deaths. Clearly, prevalence on hospitalized people in these three scenarios showed some differences in their amplitude, but all of them coincide regarding the time on lockdown decision, which definitely was a good decision. Similar information can be deduced from the number of accumulated deaths.

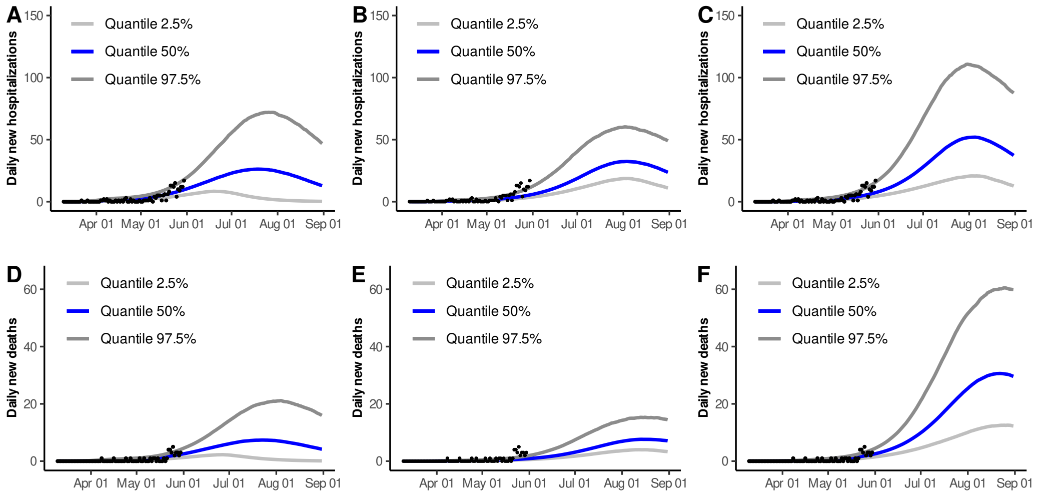

S1 Fig. 95% quantile-based intervals and median estimates for daily new hospitalizations and daily new deaths. Left, middle and right columns correspond to Scenarios 1, 2, and 3, respectively. Black dots represent available data from March 11 to May 31.

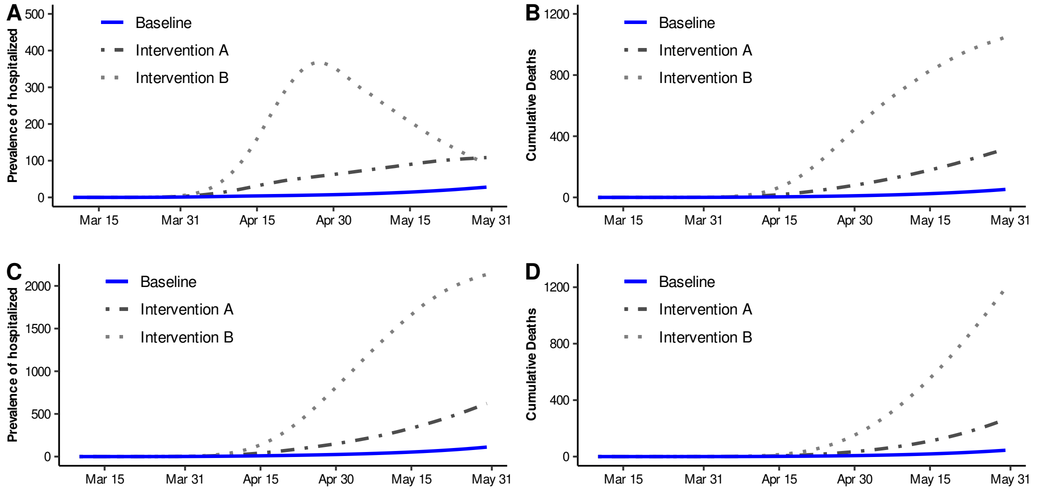

S2 Fig. Median estimates for prevalence and cumulative deaths. Blue solid line represents our baseline dynamics. Dotted-dashed line represents intervention A, that is, mitigation measures start on March 23. Dotted line represents intervention B, that is, mitigation measures start on March 30. First and second rows correspond to Scenarios 1 and 3, respectively.

S3 Possible consequences of lifting mitigation measures

S3 Fig shows possible consequences in the number of daily new cases, hospitalizations and deaths, when different proportions of individuals are released on June 01 (following the idea of Fig, 7, presented in the main document). Here, the X value considered in Scenarios 1 and 3 correspond to 12.5% and 24.91%, respectively. Observe that Scenario 1 is very similar to Scenario 2 (detailed in the main document), in the sense that an increment on the release of individuals implies an increase on the acme level. Clearly, this does not occur for Scenario 3 (S3 Fig), where there is no apparent change in the magnitude of the acme, when different amount of people is released from the protected class. This is mainly due to the median values considered in Scenario 3, for the transmission contact rates in the protected and protected released ($\tilde{\alpha}_a$ and $\hat{\alpha}_a$, respectively) classes (Table 4 in main document), which are practically the same. In this sense, there is no difference between the protected and the protected released class in this infection process. In summary, this analysis showed that data can be explained by different processes that depend on the values of the parameters, and also illustrates the difficulty of selecting the best scenario to fit the data, since more information is needed to reasonably choose model parameter values.

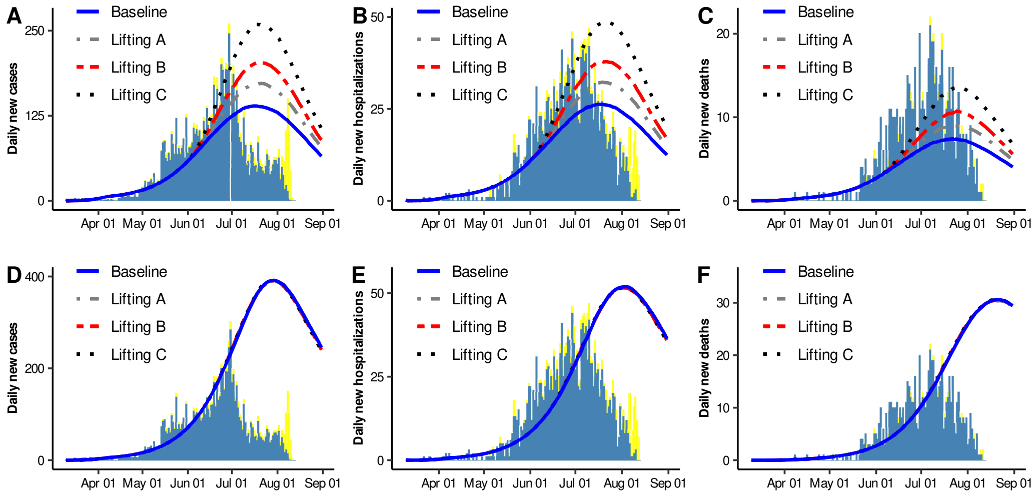

S3 Fig. Median estimates for daily new cases, daily new hospitalizations, and daily new deaths. Blue solid line represents our baseline dynamics. Grey dotted-dashed, red dashed and black dotted lines represent that approximately X%, 2X%, and 4X% of the population that fulfilled social distance measures returned to their usual activities on June 01, 2020, respectively. X values are given by each scenario. First and second rows correspond to Scenarios 1 and 3, respectively. Blue and yellow bars represent confirmed and suspected+confirmed data for Hermosillo, Sonora, Mexico.
