# Supplementary material for: Lockdown, relaxation, and acme period in COVID-19: A study of disease dynamics in Hermosillo, Sonora, Mexico
Source: PLoS One. 2020 Dec 3;15(12):e0242957. doi: 10.1371/journal.pone.0242957 (PMC7714188; doi:10.1371/journal.pone.0242957)
Supplement: Attachment — Submitted filename: renamed_b3a37.pdf [file pone.0242957.s002.pdf]

Review of the Manuscript Number PONE-D-20-28007

Lockdown, relaxation, and ACME period in COVID-19: A study of disease dynamics in Hermosillo, Sonora, Mexico

In this article, the authors develop a Kermack-McKendrick-type mathematical model in order to evaluate the confinement and relaxation measures implemented at Hermosillo (Sonora, Mexico). The phenomenon studied is of great interest and topicality, the model chosen is fairly standard, however some points deserve to be explained, such as the choice of different distributions, and also the details of the calculations.

- The introduction requires some references dealing with the mathematical and statistical study of this type of models.
- The model is quite simple, the choice of ω_{0i} must be justified, remark 1 is not sufficient.
- The choice of different distributions in a single scenario is to be justified.
- The detailed calculation for each step is necessary to validate the results obtained.
- In the initial conditions of the model, the authors say that the first case of COVID-19 recorded in Hermosillo by the Sonoran Health System, occurred on March 16, 2020 (line 128), and they consider March 11 as the start date of simulations, with the following initial condition: $I_S(0) = 1$? Where does this value come from (quote a reference)?
- At line 140, the authors assume that the first period of social distancing is $[T_{L1}, T_{U1}] = [5, 35]$ and the second period is $[T_{L2}, T_{U2}] = [50, 65]$, but between $T_{U1} = 35$ and $T_{L2} = 50$ there is no information, we need details for this period.
- In the section model parameter distributions, the authors established three different scenarios where different probability distributions were taken into account for the modeling parameters included in the mathematical model. How the authors choose the different probability distributions, I would recommend explaining their choice of distributions for the selected parameters in order to understand this part.
- In line 161 (respectively 169), the distributions for the parameters $omega_{10}$ and ω_{20} considered in scenario 3 (respectively scenario 1): $U(0.7, 0.9)$ and $U(0.1, 0.35)$ (respectively $U(0.05, 0.2)$), are not the same as those cited in table 2, why? also in the title of table 2, for a normal and uniform distribution, the parameters a and b are used, can you specify if the same parameters are used for both distributions or not.

- The general organization of the paper seems rather chaotic. In section 2 (respectively 3), the authors present the model (respectively some results), but to understand what the diagram compartment is (respectively what is the basis of these results), the reader must go to the end of the article to see the numbers. I highly recommend rearranging the paper in the usual style: each figure should be placed right after its discussion paragraph.

In conclusion, in the given form I cannot recommend the given manuscript for publication. In my opinion, a complete revision of the presentation of the content is needed.
