# Supplementary material for: Lockdown, relaxation, and acme period in COVID-19: A study of disease dynamics in Hermosillo, Sonora, Mexico
Source: PLoS One. 2020 Dec 3;15(12):e0242957. doi: 10.1371/journal.pone.0242957 (PMC7714188; doi:10.1371/journal.pone.0242957)
Supplement: Attachment — Submitted filename: Response_to_Reviewers.pdf [file pone.0242957.s003.pdf]

Blvd. Luis Encinas y Rosales S/N, Hermosillo, Sonora
C.P: 83000. Phone:
E-mail:gudelia.figueroa@unison.mx

October 29, 2020

Laurent Pujo-Menjouet, PhD
Academic Editor, PLOS ONE Journal

Dear Professor Pujo-Menjouet:

We are submitting to the PLOS ONE Journal the revised version of our manuscript entitled "Lockdown, relaxation, and acme period in COVID-19: A study of disease dynamics in Hermosillo, Sonora, Mexico". We thank the reviewers for their constructive comments, which helped us to improve this manuscript. We have revised the paper accordingly. Below, we address all comments, point-by-point, with their corresponding modifications, which can be easy tracked in the marked-up copy of the manuscript.

Regarding comments of Reviewer 1:

In this paper, the authors studied COVID-19 in Hermosillo, Mexico by using a mathematical model. The model is well-constructed and, in particular, it takes into account the effect of intervention measures by using functions w1 and w2, which represent the transition rates from susceptible to protected and protected to released, respectively. The Monte Carlo study was performed by using parameter sets based on three different scenarios, and estimation results were obtained. Based on these results, the authors discussed the effects of lockdown and lifting mitigation measures. The paper is totally very well-written and I recommend this paper for publication. The mathematical model in this paper would help the readers to evaluate situations that did not happen in reality.

Minor issues

1. Should the authors explain the meaning of the abbreviation ACME?

   Response: We really appreciate this observation since ACME, in capital letters, can be understood as an acronym. We have changed the word ACME to lower case in all the manuscript, and we also explained that acme refers to the peak of the outbreak (see page 2, line 50).

2. Should the authors estimate the (basic or effective) reproduction numbers?

   Response: Although estimation of the average new infections caused by infected people, at the beginning or during the epidemic, (throughout R0 and Rt, respectively) are of great interest in certain studies, our main objective was basically focused on the evaluation of the lockdown and relaxation measures implemented in Hermosillo, Sonora, throughout some acme characteristics. The statistical and mathematical tools used here provided enough information to reach our objectives.

Regarding comments of Reviewer 2:

In this article, the authors develop a Kermack-McKendrick-type mathematical model in order to evaluate the confinement and relaxation measures implemented at Hermosillo (Sonora, Mexico). The phenomenon studied is of great interest and topicality, the model chosen is fairly standard, however some points deserve to be explained, such as the choice of different distributions, and also the details of the calculations. In conclusion, in the given form I cannot recommend the given manuscript for publication. In my opinion, a complete revision of the presentation of the content is needed.

1. The introduction requires some references dealing with the mathematical and statistical study of this type of models.

   Response: Kermack-McKendrick mathematical models and their extensions have been widely used to study COVID-19 disease. In the first version of the manuscript, we included some references regarding to mathematical modeling of COVID-19 disease, and some others focused on the estimation of model parameters, as well as prediction and control of the dynamics of this disease. Those were included to emphasize works related to non-pharmacological interventions. We thank the reviewer for this observation. In the revised version, we distinguish those references of studies based on the Kermack-McKendrick model from a statistical perspective and add new references that show a qualitative analysis of their models [1-3] (see page 2, lines 38-40).

   New references:

   1. Ngonghala CN, Iboi E, Eikenberry S, Scotch M, MacIntyre CR, Bonds MH, et al. Mathematical assessment of the impact of non-pharmaceutical interventions on curtailing the 2019 novel Coronavirus. Mathematical Biosciences. 2020;325:108364. doi:10.1016/j.mbs.2020.108364.

   2. Okuonghae D, Omame A. Analysis of a mathematical model for COVID-19 population dynamics in Lagos, Nigeria. Chaos, Solitons & Fractals. 2020;139:110032. doi:10.1016/j.chaos.2020.110032.

   3. Bugalia S, Bajiya VP, Tripathi JP, Li MT, Sun GQ. Mathematical modeling of COVID-19 transmission: the roles of intervention strategies and lockdown. 2020;17:5961. doi:10.3934/mbe.2020318.

2. The model is quite simple, the choice of ω_{0i} must be justified, remark 1 is not sufficient.

   Response: We really appreciate this observation. In the revised version we now explain, step to step, how w_{i0} value is obtained (see pages 4-5, Remark 1).

3. The choice of different distributions in a single scenario is to be justified.

   Response:
   The probability distributions considered in each one of the scenarios, included in the manuscript, are independent proposals of different researchers and are mostly based on their respective heuristic analyses, the references they considered, as well as their experience. In some cases, a researcher selected a uniform distribution to assign an equal probability density, into an interval, to each one of the possible parameter values. On the other hand, a truncated normal distribution was selected due to its versatility, since throughout this distribution is easy to assign lower, equal, or greater probability density to certain values of model parameters. This can be observed in the following figure, when different shapes of the probability density function of a truncated normal on the interval [1.22,1.27] are obtained for different values for parameters μ and σ.

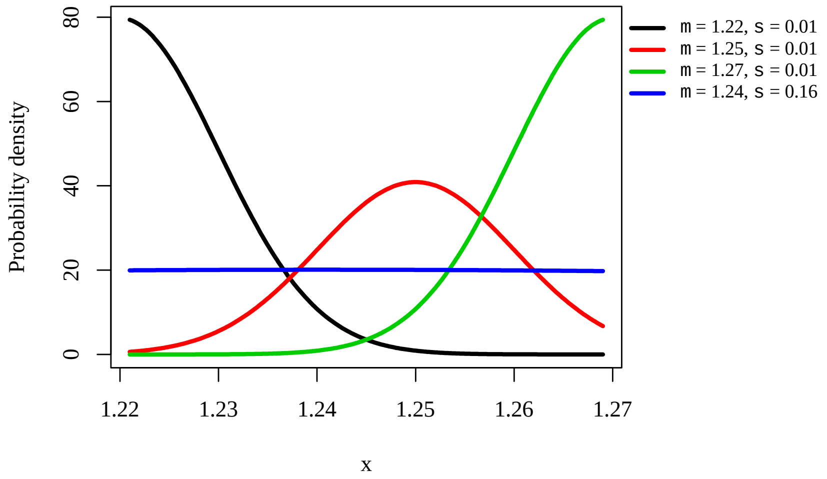

4. The detailed calculation for each step is necessary to validate the results obtained.

Response: We agree with Reviewer 2 regarding the importance of being able to validate the results we obtained. For that reason, in the revised version of the manuscript, we now provide a link to a Github repository where a detailed R file used in our calculations can be downloaded (see page 8, lines 210-217).

New reference:

1. Espíndola-Zepeda JA. R script; 2020. https://github.com/BioMatUnison/COVID-19_Proyect/tree/master/Main

5. In the initial conditions of the model, the authors say that the first case of COVID-19 recorded in Hermosillo by the Sonoran Health System, occurred on March 16, 2020 (line 128), and they consider March 11 as the start date of simulations, with the following initial condition: $I_S(0)=1$? Where does this value come from (quote a reference)?

Response: We considered March 11 as the starting date in our simulations since the first registered date by onset symptoms was March 11, according to the Hermosillo COVID-19 database. In the first version of the manuscript, we wrote down: "being March 11 the registered date of onset symptoms". Now, to clarify this point, in the revised version of the manuscript, this sentence has been replaced by: "being March 11 the onset symptoms date of the first confirmed infected case [1]" (see page 5, lines 131-132).

Reference:

1. Gobierno de México. Datos Abiertos; 2020 (accessed August 14, 2020). https://www.coronavirus.gob.mx/datos/.

6. At line 140, the authors assume that the first period of social distancing is $[T_{L1},T_{U1}]=[5,35]$ and the second period is $[T_{L2},T_{U2}]=[50,65]$, but between $T_{U1}=35$ and $T_{L2}=50$ there is no information, we need details for this period.

Response: Thanks for the observation. The objective of considering the periods $[T_{L1}, T_{U1}] = [5,35]$ and $[T_{L2}, T_{U2}] = [50,65]$ is to model the processes of confinement and breaking confinement, respectively. These processes follow the dynamics given by equations 2 and 3. These equations are step functions, and for our dynamics, take a value equal to zero outside the range of interest. Hence, for the interval $(T_{U1}, T_{L2}) = (35, 50)$, both equations take value equal to zero (see page 5, lines 146-148).

7. In the section model parameter distributions, the authors established three different scenarios where different probability distributions were taken into account for the modeling parameters included in the mathematical model. How the authors choose the different probability distributions, I would recommend explaining their choice of distributions for the selected parameters in order to understand this part.

   Response: We consider that response provided in question 3, already answers this observation. A paragraph is now included in the main manuscript to clarify this issue, to address this useful comment (see page 6, lines 157-163).

8. In line 161 (respectively 169), the distributions for the parameters $\ omega_{10}$ and ω_{20} considered in scenario 3 (respectively scenario 1): $U(0.7,0.9)$ and $U(0.1,0.35)$ (respectively $U(0.05,0.2)$), are not the same as those cited in table 2, why? also in the title of table 2, for a normal and uniform distribution, the parameters a and b are used, can you specify if the same parameters are used for both distributions or not.

   Response: The $U(0.7,0.9)$ and $U(0.1,0.35)$ distributions in Scenario 3 were assigned to the protected proportion of susceptible individuals and to the proportion of protected individuals that breaks the confinement, respectively. Something similar was considered in Scenario 1, using $U(0.7,0.9)$ and $U(0.05,0.2)$ distributions. Although these uniform distributions were not assigned to ω_{10} and ω_{20}, they were used to set their respective distributions, as is explained in the main manuscript (see pages 6-7, lines 170-187). We believe that modifications implemented in *Remark 1* will help clarifying this point.

   On the other hand, thanks for the observation regarding the use of parameters a and b in two different distributions. In the revised version of the manuscript, a and b are used for a truncated normal distribution and c and d for a Beta distribution (see caption of the Table 2, page 6).

9. The general organization of the paper seems rather chaotic. In section 2 (respectively 3), the authors present the model (respectively some results), but to understand what the diagram compartment is (respectively what is the basis of these results), the reader must go to the end of the article to see the numbers. I highly recommend rearranging the paper in the usual style: each figure should be placed right after its discussion paragraph.

   Response: PLOS ONE's submission of a manuscript proposal requires not including figures in the pdf file. These must be uploaded as separate files. That explains the format of the manuscript you received for revision.

Sincerely,

Dr. Gudelia Figueroa P.
University of Sonora, Mathematics Department.
gudelia.figueroa@unison.mx
